# Exercise Training as Therapeutic Approach in Cancer Cachexia: A Review of Potential Anti-inflammatory Effect on Muscle Wasting

**DOI:** 10.3389/fphys.2020.570170

**Published:** 2021-02-04

**Authors:** Luana G. Leal, Magno A. Lopes, Sidney B. Peres, Miguel L. Batista

**Affiliations:** ^1^Integrated Group of Biotechnology, Laboratory of Adipose Tissue Biology, University of Mogi das Cruzes, Mogi das Cruzes, Brazil; ^2^Technological Research Group, University of Mogi das Cruzes, Mogi das Cruzes, Brazil; ^3^Laboratory of Metabolism of Bioactive Lipids, Institute of Physiology of the Czech Academy of Sciences, Prague, Czechia; ^4^Department of Physiological Sciences, State University of Maringá, Maringá, Brazil

**Keywords:** muscle wasting, therapeutic exercise, resistance, aerobic, muscle atrophy, systemic inflammation, tumor, neoplasms

## Abstract

Cachexia is a multifactorial inflammatory syndrome with high prevalence in cancer patients. It is characterized by a metabolic chaos culminating in drastic reduction in body weight, mainly due to skeletal muscle and fat depletion. Currently, there is not a standard intervention for cachexia, but it is believed that a dynamic approach should be applied early in the course of the disease to maintain or slow the loss of physical function. The present review sought to explain the different clinical and experimental applications of different models of exercise and their contribution to a better prognosis of the disease. Here the advances in knowledge about the application of physical training in experimental models are elucidated, tests that contribute substantially to elucidate the cellular and biochemical mechanisms of exercise in different ways, as well as clinical trials that present not only the impacts of exercise in front cachexia but also the challenges of its application in clinical practice.

## Introduction

Cachexia (from the Greek *kakos* means “bad,” and *hexis* means “condition”) is a complex, multifactorial syndrome, with the etiology still unknown; its main characteristic is the involuntary and progressive loss of lean and fat mass (Argiles et al., 2003; Fearon et al., 2012; Blum et al., 2014). In addition, the presence of systemic inflammation, insulin resistance, and anorexia are closely associated with the development of cachexia syndrome, contributing to the increased patient morbidity and debilitation (Inui, 2002).

Cachexia is associated with different types of chronic conditions, such as chronic obstructive pulmonary disease, heart failure, septicemia (Argiles et al., 2010), chronic kidney disease (CKD) (Kir et al., 2016), acquired immunodeficiency syndrome (AIDS) (Tisdale, 2009), and cancer. About 50–80% of cancer patients suffer from cachexia, depending on the type and location of the tumor mass, progressing considerably in advanced stages of the disease (Inui, 2002; Tisdale, 2009). In this sense, the degree of severity and prevalence of cachexia is often associated with the type of cancer, with gastrointestinal and pancreatic cancers being the most prevalent, reaching more than 85% of cases (Tisdale, 2009). The high prevalence of cachexia in cancer patients contributes to the high morbidity and mortality rate, since cachexia may be directly responsible for 20% of deaths (Vagnildhaug et al., 2017).

**FIGURE 1:**
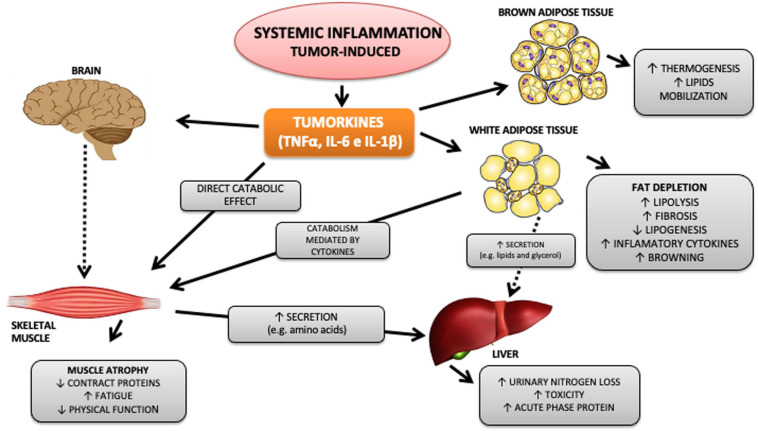
Tumor-induced systemic inflammation and metabolism impact. Among the numerous cytokines released by the tumor, TNFα, IL-6, and IL-1β stand out. These, in turn, are capable of acting on different organs and systems, promoting inflammation, metabolic and immunological changes that directly and indirectly affect essential organs in the pathophysiology of cachexia, such as skeletal striated muscle and white and brown adipose tissue. Continuous arrows refer to mechanisms already described, and dotted arrows refer to mechanisms not yet known.

Currently, cancer cachexia (CC) is directly associated with systemic inflammation and is characterized as a chronic inflammatory syndrome (Fearon et al., 2006; Evans et al., 2008; Batista et al., 2012b; Argiles et al., 2014) elicited by the interaction between the tumor and the host, which triggers a subsequent immune response. As a result, there is an increased infiltration of cells of the immune system in affected tissues, especially in adipose tissue (Henriques, 2020) and skeletal muscle (Anoveros-Barrera et al., 2019), and consequent release of tumor secreted products, mainly TNFα and IL-6, among others (Tsoli and Robertson, 2013). The release of inflammatory factors alters the homeostasis of various organs and tissues, such as; (1) Hypothalamus, which promotes the reduction of food intake, (2) liver, inducing the production of hepatic acute phase proteins, which repress drug clearance pathways, resulting in higher toxicity of anticancer agents, (3) skeletal muscle, promoting an imbalance between anabolic and catabolic contractile proteins, leading to reduced muscle mass, increased fatigue, and sarcopenia, and (4) brown (TAM) and white (TAB) adipose tissues, increasing lipolysis, reducing adipogenesis (Franco et al., 2017), lipogenesis, and thermogenesis (Tsoli and Robertson, 2013; Figure 1).

The maintenance of skeletal muscle mass is reached by a balance between protein synthesis and degradation, which in turn, is mainly regulated by physiological inputs such as the nutritional status and exercise training (Gallagher et al., 2012). Recent studies have focused on the ubiquitin-proteasome pathway, regulation of satellite cells in skeletal muscle, and the importance of related receptors and signaling pathways that are likely to be influenced by tumor-induced systemic inflammation (Fearon, 2011). Lean body mass loss contributes to a progressive weakening in muscle strength and endurance (Gallagher et al., 2012), exercise capacity (England et al., 2012; Jones et al., 2012b) physical activity levels (Wilcock et al., 2008; Dodson et al., 2011), and poor survival (Antoun et al., 2013; Martin et al., 2013). Approaches that aim at increasing increase synthesis or reducing the degradation of muscle proteins, or both, can positively contribute in order to limit or reverse the reduction of muscle mass in patients with CC (Maddocks et al., 2012).

For this purpose, resistance exercise provides a potent stimulator of muscle protein synthesis, and this is mainly confirmed when performed in conjunction with supplementation of branched-chain amino acids (Hakkinen et al., 1998). Although increased muscle proteolysis is also observed immediately after resistance exercise, protein synthesis is stimulated to a greater degree, which provides a positive protein balance resulting in a higher muscle protein content (Schoenfeld, 2010).

It is also noteworthy that the regular practice of exercise, organized in a training program (exercise training), exerts anti-inflammatory effects, which would lead to protection against chronic inflammatory conditions, notably by the reduction of pro-inflammatory cytokines and C reactive protein levels (Petersen and Pedersen, 2005; Fischer, 2006; Lira et al., 2009a; Batista et al., 2010). However, the exact mechanisms of this “beneficial effect,” still remain to be elucidated. It is well established that skeletal muscle plays an important role as an immunogenic organ and an important mediator of the anti-inflammatory response (Petersen and Pedersen, 2005; Pedersen et al., 2007; Pedersen and Fischer, 2007). In this scenario, exercise-induced muscle contraction increases interleukin-6 (IL-6) gene expression and secretion by skeletal myocyte, according to the diversity of exercise performance variables (volume, intensity, duration), and an increase in plasma IL-6 levels (Leal et al., 2018). Also, induced by IL-6 (Steensberg et al., 2003a), a subsequent increase in other cytokines as interleukin-10 (IL-10), ILra and soluble tumor necrosis factor I and II receptors (TNF I and II) occurs. The complex secretion of several cytokines is characterized as an “anti-inflammatory effect,” a condition observed after an acute session of aerobic exercise (Petersen and Pedersen, 2005).

**FIGURE 2:**
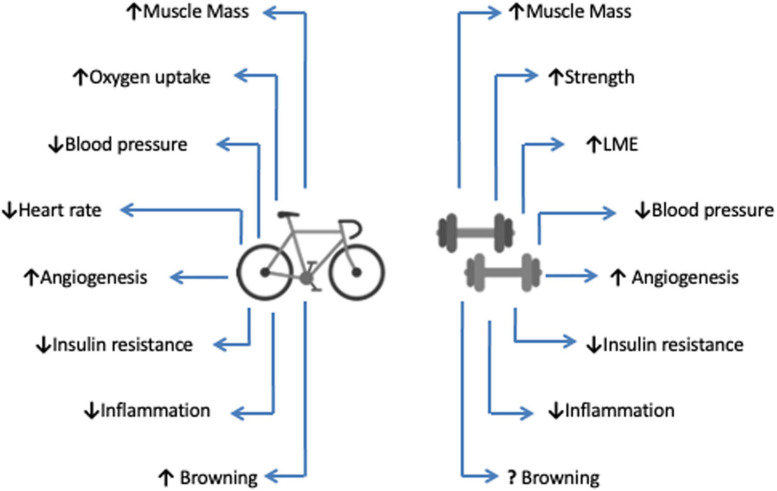
Main physiological effects of aerobic and anaerobic exercise training. Both types of exercise. Both different exercise types promote numerous physiological changes that result in improved physical capacities and health indicators, resulting in increased life expectancy (survival). Aerobic exercise is represented by the bicycle, and the dumbbells describe anaerobic exercise. LME, Local Muscular Endurance.

In fact, exercise training may attenuate the effects of CC via diverse mechanisms, including the modulation of muscle metabolism, insulin sensitivity, and levels of inflammation (Maddocks et al., 2012). Although the many benefits of exercise set a beneficial scenario as an intervention to cachexia symptoms, the lack of studies on the direct evaluation of its effects still raises major questions. Therefore, the present review seeks to elucidate the mechanisms of action of different modalities of exercise training in view of the main symptoms of CC (Figure 2). In particular, emphasis will be placed on the overall anti-inflammatory effect of exercise training on muscle wasting, in addition to the possible repercussions on survival during CC.

## Exercise as Therapeutic Strategy

CC syndrome, as mentioned earlier, is characterized by a progressive weight loss in cancer patients, a condition that interferes with treatment outcomes and directly affects a patient’s quality of life (Fearon et al., 2011). In such condition, nowadays, what it is becoming increasingly appreciated at the moment is to find out how the combination of anti-tumor and anti-cachexia therapies, i.e., a synergistic approach would increase the response rates and survival (Laviano et al., 2005).

Currently, there are no standard treatments for cachexia, but recommendations emphasize greater emphasis on the application of a proactive approach in the initial phase of the disease with the intention of maintaining physical fitness or decreasing its rate of decline (Maddocks et al., 2011). In 2011, the consensus published by Fearon et al. (2011) aimed at developing a framework for the definition and classification of CC (syndrome classification according to the stage of evolution and level of involvement), and the most common strategies suitable for treatment at different stages of cachexia (Pre-Cachexia, Cachexia and Refractory Cachexia), as follows: (1) pre-Cachexia stage: patient monitoring and preventive treatment are indicated; (2) cachexia stage: multimodal management according to the presented phenotype, prioritizing contributing factors in order to reverse the picture; (3) refractory cachexia stage: palliative treatment of symptoms, psychosocial support and ethical discussion about nutritional support are indicated (Fearon et al., 2011).

In the last few years, multimodal treatment has been the most recommended therapeutic approach as an adjuvant intervention in CC. In this sense, the main multimodal components are; (1) multi and interdisciplinary care; (2) treatment of secondary consequences of cachexia; (3) pharmacotherapy directed to inflammatory processes and metabolic alterations; (4) nutritional counseling; (5) physical therapy and exercise training; and (6) social and psychological support (Arends et al., 2017; Naito, 2019). The multimodal treatment has been the most recommended therapeutic approach since CC diagnosed comprises: (1) multi and interdisciplinary care; (2) treatment of secondary consequences of cachexia; (3) pharmacotherapy directed to inflammatory processes and metabolic chaos; (4) nutritional counseling; (5) physical therapy and physical exercise; and (6) social and psychological support (Grande et al., 2015; Arends et al., 2017).

The incorporation of exercise training as a therapeutic strategy should be applied preferentially in the first two stages of the syndrome, pre-cachexia and cachexia, thus attenuates muscle wasting and inflammation. It is noteworthy that in clinical practice, not only the diagnosis, but also the proper classification of the patient between the stages is puzzling, which makes the clinical approach challenging. The lack of proper use of criteria for the diagnosis and staging of cachexia, makes it difficult to determine its prevalence and impact and to assess whether interventions might be adopted in cachectic populations without formal recognition, as the case with exercise studies, particularly in advanced cancer (Grande et al., 2014).

Exercise attenuates the CC effects through different mechanisms, including increased anabolism or maintenance of skeletal muscle homeostasis, improved insulin sensitivity and control of inflammation levels (Grande et al., 2014). Therefore, the implementation of an adequate exercise program combined with an adequate and structured diagnosis according to the treatment stage provides the possibility of reversing protein degradation and stimulates protein synthesis, which guarantees the maintenance of lean mass, ameliorating muscle wasting observed in CC (Gould et al., 2013). Inflammation control is also of the utmost importance in CC as reported in animal and human studies (Seelaender and Batista, 2014).

It has even been proposed that a ratio between pro- and anti-inflammatory cytokines may serve as a marker of CC severity of Argiles et al. (1997). For example, IL-10 (anti)/TNFα (pro) ratio has been adopted as a marker of inflammation level in obese individuals and animals (Jung et al., 2008). The increase in pro-inflammatory state is closely related to the decrease in survival and morbidity in CC (Lira et al., 2009c). Therefore, exercise training, either through the Resistance Training (RT) or endurance training, seems to contribute satisfactorily to the control of inflammation by modulating the balance of the main pro and anti-inflammatory cytokines (TNFα, IL-6, and IL-10) (Donatto et al., 2013).

### Exercise Training as a Modulator of Inflammation

In CC, systemic inflammation may have various origins such as tumor cells themselves as well as activation of immune cells that release cytokines, chemokines, and other inflammatory mediators (Argiles et al., 2014). Although the tumor appears to be primarily responsible for the activation of immune cells, the intestine also plays an important role in this process due to the fact that intestinal barrier dysfunction and bacterial translocation are associated with cancer (Klein et al., 2013). Numerous inflammatory cytokines, such as tumor necrosis factor α (TNFα), IL-6, and interleukin-1 (IL-1), are elevated in cachectic cancer patients and are known to promote catabolism (Argiles et al., 2012a). Although different cancer cells may release distinctive cytokines, the circulating levels from different host tissues appears increased as a response to cancer (Argiles et al., 2009). Multiple humoral factors and cellular signaling pathways appear to be involved in the reduction of cancer-derived muscle mass, which worsens the patient’s prognosis. It is believed that systemic inflammation may be the main mediator of this process, and, therefore, unveiling its real origin in cancer patients may contribute to the development of an effective therapy (Zhang et al., 2017).

Among the different types of treatment that aim at reversing or restricting the progression of CC is the use of pharmacological interventions with anti-cachexia agents and the treatment of inflammatory cytokines designed to contain cachexia, especially TNFα (Gould et al., 2013). Among the various non-pharmacological strategies that have been investigated, different modalities of exercise training, such as endurance, resistance training, and combined training appear to act favorably in controlling inflammation, as they are capable of inducing an increase in anti-inflammatory cytokine secretion by adipose tissue causing significant reduction in C-Reactive Protein (CPR) levels (Lira et al., 2009c; Pierce et al., 2009; Galvao et al., 2010).

As already mentioned, multiple inflammatory cytokines such as TNFα, IL-6, and IL-1 are known to promote muscle wasting during CC progression (Zhang et al., 2017). However, it is well characterized that after an exercise session, there is an exponential increase in the levels of IL-6 (more than 100 times) acutely, which is dependent on the configuration of the exercise variables, such as intensity, duration, amount of muscle mass recruited and individual aerobic capacity (Fischer et al., 2003; Steensberg et al., 2003b; Fischer, 2006). It has been observed that administration of IL-6 at levels similar to those observed in the plasma of subjects following long-term and high intensity exercise is capable of promoting significant reductions in TNFα levels (Starkie et al., 2003). Such evidence is corroborated by studies that evaluate the rates of pro-inflammatory cytokines in animals with induced cancer undergoing exercise training. Interesting, High-Intensity Interval Training (HIIT) efficiently increases survival rate and reduces tumor mass growth in tumor-bearing mice (Alves et al., 2018). Similar data can be found in a study that evaluated the effects of voluntary running against tumor progression and survival of animals with different tumor types. Also, a positive correlation was observed between increased cytokine expression (especially IL-6) by tumor cells and activation and redistribution of Natural Killer cells, which in turn is directly responsible for suppressing tumor growth by up to 60% (Pedersen et al., 2016).

Adipose Tissue (AT) is deeply affected during CC and might play an important role in systemic inflammation (Batista et al., 2012a). For instance, epididymal AT presents an early impairment of lipid synthesis and storage, which is not observed in the mesenteric area, more resistant to the “fat reduction effect” of CC more than any other depot. In addition, an increase in the size of adipocytes and a consequent increase in the production of pro-inflammatory proteins, such as tumor necrosis factor alpha (TNFα) and membrane receptors (TNFR1 and TNFR2) (Figueras et al., 2005) are reported. On the other hand, endurance training is able to induce a higher production of IL-10 from AT, an important anti-inflammatory cytokine, which contributes to modulate TNFα secretion and control inflammation (Lira et al., 2009b).

The anti-inflammatory effect of exercise is more evident in certain pathological conditions, such as atherosclerosis, type II diabetes mellitus, obesity and chronic heart failure (CHF), conditions characterized as low-intensity chronic systemic inflammation and by a twofold to a threefold systemic increase in pro-inflammatory cytokine and C-reactive protein levels (Fischer et al., 2003).

Inflammatory cytokine levels when measured in the AT of animals with induced CC and submitted to RT is deeply modulated. The levels of IL-10, TNFα, and IL-6, and the ratio between IL-10 and TNFα are systemically up-regulated, but the levels of IL-6 in AT are down-regulated. These findings were followed by a reduction in tumor volume and the preservation of muscle mass (Donatto et al., 2013). Endurance training also improves the IL-10/TNFα ratio and induces a reduction in monocytes infiltration into different visceral adipose tissue depots (mesenteric and retroperitoneal) of tumor-bearing rats (Lira et al., 2012; Table 1).

**TABLE 1:** Studies published in the last 10 years with experimental rat and mouse models.

**Author**	**Animal/culture**		**Cachexia markers**	**Exercise training protocol**			**Main results**
	**Species/lineage**	**Induction method**		**Type**	**Protocol**	**Volume/intensity**	
Ballaro et al. (2019)	Female BALB/c mice	5 × 10^5^, C26 tumor cells	↓ Total body mass ↓ Muscle mass ↓ Adipose tissue mass	Aerobic training	Moderate exercise on treadmill	Running speed by 11 m/min for 45 min	• Exercise-induced a decrease in muscle autophagy markers • Exercise improves mitochondrial mass and activity in skeletal muscle • Exercise reduces muscle gene expression of MuRF1 and Atrogin-1
Ranjbar et al. (2019)	Balb/c mice	5 × 10^5^ C26 carcinoma cells	↓Body weight ↓Muscle weight ↓Functional capacity	Combined training	1° Resistance Training (ladder protocol) and; 2° aerobic training (motorized Wheel), on the same day	4 days/week, 4 weeks before and 11 days after tumor implantation RT - 3 sets of 2 repetitions at 50% of animal body weight AT - 25 min. 5–9 m/min speed	• Exercise does not restore body weight loss • Exercise does not prevent gastrocnemius loss but increase tibialis anterior weight • Training was able to reduce autophagy markers in muscle • Exercise does not decrease atrophy markers
Alves et al. (2018)	C57BL/6	10^6^ LLC cells	No control group was presented to indicate presence of cachexia	High-intensity interval training (HIIT)	Treadmill running	Each session consisted of 5 intervals of 3 min running at 18 m min^–1^ followed by 4 min running at 25 m min^–1^	• Decrease on tumor size in tumor trained group • Increase on PDL1 and VEGF expression in tumor • Increase of survival
Das Neves et al. (2016)	Wistar rats	10^6^, Walker 256 tumor cells injected in the bone marrow	Muscle atrophy, inflammatory markers (TNFα)	Resistance training (RT)	Hind limb (i.e., “squat-like” movement). Started after tumor injection with daily sessions for 15 days	1–3 sets; 10–15 rep; 65% 1RM	• Cancer-induced not effect on total body mass • Muscle atrophy with no changes in RT group • No difference in atrophy markers • Loss of strength associated with decrease on survival
Khamoui et al. (2016)	Female Balb/c mice	5.0 × 10^5^, C26 tumor cells	↓ Total body mass, muscle and fat mass ↓ Physical function	Resistance and aerobic training	Training started 8 weeks before tumor injection and during 3 weeks after tumor injection RT was performed on a climb ladder AT in a motorized wheels	• RT—3 days per week, 5 sets of 3 repetitions with progressive loads • AT—60 min, 5 days per week. Speed between 5–6.5 m/min	• RT and AT not restore total and lean or fat mass in C26 group •↑ In IGF-1 gene expression in muscle of RT-C26 and AT-C26 •↑ In Myogenin gene expression in muscle of RT-C26 group • No differences in physical function in trained groups
Pigna et al. (2016)	Female BALB/c mice	Grafting of 0.5 mm^3^ fragment of colon carcinoma (C26)	↓ Total body mass ↓ Muscle mass	Aerobic training	Wheel-running activity	Running for 20:59 ± 4:30 (h min) a day at Speed by 2.1 ± 0.1 (km/h)	• Increase in life span in wheel running C26-bearing mice • Exercise-induced muscle hypertrophy in C26-bearing mice • Exercise-induced a decrease in muscle autophagy markers • Exercise reduces muscle gene expression of MuRF1 and Atrogin-1
Pedersen et al. (2016)	Female C57BL/6 and NMRI-Foxn1nu mice	2 × 10^5^ B16F10 melanoma cells 2 × 10^5^ LLC tumor cells	↓Body weight ↑Inflammation	Running wheels	Voluntary running, started 4 weeks prior to tumor cell inoculation	Only total running distance was evaluated	• Running decrease tumor growth • Upregulation of pathways associated with immune function • Levels of epinephrine and IL-6 are related to tumor growth through mobilization of NK cells
Pin et al. (2015)	Balb/C or C57BL/6	5 × 10^5^ Colon26 or LLC cells	↓ Muscle mass ↓ Physical function ↓Hematocrit content	Aerobic training	Treadmill Running, 5 days/week, 14 or 28 days after C26 or LLC implantation, respectively	Mice ran for 45 min at the speed of 14 m/min	• In the C26 hosts, acute exercise does not prevent muscle wasting • LLC hosts are responsive to exercise and their treatment with the EX-EPO combination prevents the loss of muscle strength • LLC EX-EPO group increases muscle oxidative capacity
Donatto et al. (2013)	Wistar rats	Walker 256, 3 × 10^7^ tumor cells	↓ Total body mass ↓ Muscle mass inflammatory markers	Resistance training	8 weeks of resistance training, with climbing sessions	Training sessions were 3–5 ladder climbs with 75, 90, and 100% of the rat’s previous maximal carrying capacity	• RT increased by 9% body weight gain in TB group • LDL-c levels were decreased with RT in TB group • HDL-c levels were increased with RT in TB group • IL-10/TNFα ratio was higher with RT in TB group • RT attenuate the protein content of IL-6
Lira et al. (2012)	Wistar rats	Walker-256 carcinosarcoma (2 × 10^7^ cells/rat)	↓Body weight	Aerobic training	8 weeks of treadmill running	15–60 min at 10 m/min. Intensity maintained between 60 and 65% VO2 max	• Training groups showed decrease on adipose tissue content; • Decrease on tumor size in tumor trained group • Exercise tumor group showed decrease of IL-6 MEAT content • Exercise tumor group showed decrease on IL-10/TNFα ratio in MEAT group • Decrease of macrophages infiltration on adipose tissue with training
Penna et al. (2011)	C57BL/6 mice	5 × 10^5^ LLC cells	↓ Muscle mass ↓ Adipose tissue mass	Aerobic training	Treadmill running	14 m/min, 45 min, 5 days/week	• Exercise + EPA treatment attenuate muscle wasting • Exercise + EPA improve muscle strength • Exercise + EPA reduce tumor weight

*TNFα, Tumor Necrosis Factor-Alpha; RM, Maximal Repetition; RT, Resistance Training; AT, Aerobic Training; IGF-1, Insulin-Like Growth Factor-1; C26, Colon-26 Adenocarcinoma; MURF-1, Muscle RING-Finger Protein-1 (Murf1); ATROGIN-1, Muscle-Specific Ubiquitin Ligase; EPA, Eicosatetraenoic Acid; LLC, Lewis Lung Carcinoma; TB, Tumor Bearing Group; LDL, Low Density Lipoproteins; HDL, Hight Density Lipoproteins; IL-6, Interleukin Type 6; NK, Natural Killer Cells; PDL-1, Programmed Death-Ligand 1; VEGF, Vascular Endothelial Growth Factor.*

Few studies in clinical practice, to date, have investigated the systemic effects of training on the inflammatory profile during CC (Lira et al., 2012; Das Neves et al., 2016). With this limited range of information, it becomes complex to extrapolate outcomes to cancer patients, especially at an advanced stage. To this end, further studies to explore the effects of training on both systemic and local inflammation markers are required.

### Exercise Training and Muscle Atrophy in Cancer Cachexia

Muscle wasting is one of hallmarks of CC (Fearon et al., 2011; Fearon, 2011; Bedard et al., 2015). During CC, muscle protein breakdown occurs disproportionately compared to other tissues, causing widespread weakness and debilitation. Once respiratory muscles are affected asphyxiation and death may take place (Cai et al., 2004).

Three systems are described for acting on protein degradation in skeletal muscle: the lysosomal system; the activated calcium system; and the ubiquitin-proteasome system (UPS), which degrades most cellular proteins (Tisdale, 2009). The latter is an ATP dependent process of labeling protein substrates with ubiquitin molecules, mediated by Ubiquitin E3 Ligases (Bilodeau et al., 2016).

It has been observed in different studies that moderate-intensity running is able to stimulate muscle hypertrophy, reduce autophagy markers, increase mitochondrial activity and reduce gene expression of Murf and Atrogin-1 Ubiquitin ligases in the muscle of BALB/c mice with C26-induced cachexia (Pigna et al., 2016; Ballaro et al., 2019). However, when combined with climbing (combined training) the total mass reduction is not inhibited, but only the activity of autophagy markers without altering the UPS system (Ranjbar et al., 2019; Table 1).

Different mechanisms are involved in reducing muscle mass during CC. Several hormones, cytokines and tumor-derived factors have been shown to influence protein balance in normal and disease situations through various intracellular signal transduction systems (Zhou et al., 2010). While the IGF1/PI3K/AKT pathway is related to muscle protein synthesis, an opposite pathway mediated by activation of FOXO transcription factors as well as the NF-κB pathway in conjunction with Smad transcription factors promote protein breakdown (Glass, 2005).

Myostatin, a member of the TGFβ growth factor superfamily, is considered a negative regulator of skeletal muscle mass under many conditions of muscle loss (Smith and Lin, 2013) detected at high levels during the embryonic and postnatal developmental stages (Argiles et al., 2012b). Myostatin deletion increases muscle regeneration through satellite cell activation and self-renewal, thus promoting postnatal muscle growth and repair (Mccroskery et al., 2005). Several different types of myostatin pathway inhibitors, including anti-myostatin antibodies and their receptor (ActRIIB), are under clinical development. Preliminary results point to the preservation of muscle mass in patients with muscular dystrophy and cancer (Lee et al., 2005; Zhou et al., 2010).

In addition, myostatin works to inhibit muscle growth and regeneration by regulating MyoD (myogenic differentiation factor) expression, resulting in reduced muscle mass. MyoD is a direct target of the NF-κB transcription factor activation cascade that is activated during cachexia by TNFα (Guttridge et al., 2000). Considering NF-κB as a potent inhibitor of MyoD expression, there is a possibility that myostatin could signal via NF-κB to regulate MyoD expression (Mcfarlane et al., 2006; Coletti et al., 2016).

The mechanism involved in muscle protein degradation during LLC-induced cachexia has been addressed by Zhang et al. (2017). Co-culture of C2C12 myotubes in LLC cell-treated medium, rapid activated catabolic response in a TLR4-dependent manner, including activation of the p38 MAPK-C/EBPβ signaling pathway, as well as ubiquitin-proteasome and autophagy pathways resulting in myotube atrophy. In contrast, TLR4 knockout animals with induced CC showed muscle mass preservation and lower levels of TNFα and IL-6 compared to wild-type animals. Taken together, the data set suggests that TLR4 is the central mediator of muscle atrophy observed in CC and also an important therapeutic target.

Toll-like receptors (TLRs) are highly conserved transmembrane proteins that play an important role in the detection and recognition of microbial pathogens (Lu et al., 2008). TLR4 acts as a lipopolysaccharide (LPS) receptor, and associates with myeloid differentiation protein 2 (MD2) to form a complex to interact with LPS (Henriques et al., 2018). Both acute and chronic endurance aerobic exercise are cited for decreasing TLRs expression on the monocyte cell surface, also decreasing inflammatory cytokine production and TLR4 cell surface expression in monocytes. These effects are related to post-exercise immunosuppression and are responsible for increasing athletes’ susceptibility to infection (Gleeson et al., 2006).

Few studies are investigating the impact of exercise training on Toll-like receptors. It was shown that 1.5 h of cycling (∼65% VO2 max) in heat (34°C) acts on TLR expression and function *in vivo* (healthy men), followed by a reduction in post exercise (immediately after the end of session) TLR1, TLR2 and TLR4 expression and after 2 h of recovery (Lancaster et al., 2005). The acute effects of eccentric resistance training on the TLR4 signaling pathway were evaluated in men who underwent two eccentric RT sessions at 6 weeks intervals, with 1 group continuing the training and while the other remained at rest. Acute eccentric RT increased TLR4-mediated NF-κB and MAPK activation and TNFα levels in human peripheral blood mononuclear cells, suggesting a pro-inflammatory response. However, the 6 weeks RT group reduced TLR4-mediated activation of the pro-inflammatory response via independent and MyD88-dependent pathways (Fernandez-Gonzalo et al., 2012).

NF-κB has been considered a pro-inflammatory signaling pathway activated by pro-inflammatory cytokines such as IL-1 and TNFα (Lawrence, 2009). NF-κB in the muscle is activated by disuse or sepsis and plays an important role in the pathogenesis of these conditions, however, there are other alternative intracellular pathways, such as caspases and JNK/AP-1, which are also capable of being activated by cytokines (Cai et al., 2004). NF-κB blockade is able to inhibit protein catabolism in C2C12 myotubes (Li and Reid, 2000). However, although the mechanisms of action of NF-κB are established in innate immunity, inflammation and apoptosis, the role of this transcription factor in the muscle is not yet fully understood.

As already mentioned, intense exercise acutely promotes a significant inflammatory response. In RT practitioners, after an intense training session, IκBα muscle protein levels decreases (a member of a family of NF-κB inhibiting cellular proteins), while p-NF-κB (p65) protein levels increase by 2 h after exercise and return to near-basal levels within 4 h of exercise. In addition, it has been observed that both the circulating levels and the MCP-1, IL-6 and IL-8 mRNA are up-regulated significantly 2 h after exercise. These findings indicate that intense resistance exercise transiently activates NF-kB signaling in human skeletal muscle during the first hours after exercise (Vella et al., 2012). Another study has investigated the effects of endurance exercise to exhaustion in trained rats finding that RT increases p50, IκBα, and phosphor-IKK kinases content in muscles. Moreover, temporal analysis indicates that corroborating the previous study, higher levels of NF-κB ligands were observed 2 h after exercise, while p65 reached maximum levels between 2 and 4 h. The authors suggest a link between increased muscle activation of NF-κB to exercise redox factor (Ji et al., 2004).

In summary, systemic inflammation is the main cause of muscle atrophy and fatigue in CC, clinical marks also described in the aging process, AIDS, chronic heart failure, COPD and also in CC (Li et al., 2008). On the other hand, RT present itself as an alternative type of training capable of modulating CC independently of drugs, mainly due to its ability to promote an anti-inflammatory scenario.

## Exercise and Tumoral Progression

The first published paper on the effects of exercise on tumor growth was conducted in 1944 (H. P. Rusch and Kline, 1944). The influence of TR on the rate of tumor growth in mice transplanted with fibrosarcoma was investigated; in this case, the animals preserved weight loss and the rate of tumor growth was also lower than that observed in the control group. As a consequence, a precedent was opened for numerous reports seeking to elucidate the mechanisms involved.

Another study, published in 1962, sought to evaluate the effects of combined high intensity running-swimming training, both performed to fatigue, on Wistar rats inoculated with Walker 256 tumor. The results showed that tumor weight and size in control animals were greater compared to the trained groups. There were even cases of complete tumor regression in the exercised animals (Hoffman et al., 1962). In another trial performed in the same work one group received an injection of an inhibitory substance called (F-Substance) produced by fatigued muscles (muscles were suspended with a 0.85% NaCl bath and electrically stimulated to “fatigue”). F-substance simulate the effects of exercise and assessed whether it would inhibit tumor growth in a similar way to RT. The results were compatible with those found in the trained group; however, authors did not investigate the components of the substance or its mechanism of action (Hoffman et al., 1962).

Nonetheless, two recent studies using Walker 256 tumor-induced cachexia that performed RT (climbing training) present divergent data on tumor volume. One showed that the trained animals did not undergo any changes in tumor mass compared to their matched control; however, muscle mass was preserved and inflammatory markers such as TNFα and IL-6 were reduced in the plasma of animals submitted to RT (Donatto et al., 2013). The other study showed a 10% reduction in tumor volume (*p* < 0.05) (Bacurau et al., 2007), and lower circulating levels of inflammatory markers. However, a study evaluating the effects of two different types of training, aerobic and RT training, on induced cachexia in Balb/c mice found that the animals that practiced RT presented a tumor volume increase of about 30% (Khamoui et al., 2016).

A study evaluating the effects of voluntary running on animals with CC induced by different tumor types found a positive regulation of both pro- and anti-inflammatory cytokines (IL-6, IL-1β, TNFα, iNOS), intense mobilization and trafficking of NK cells (natural killer) and lower incidence and reduction of tumor volume by up to 60% (Pedersen et al., 2016). Also, CD-68 expression (expressed in monocytes, used as a marker for macrophages), NKp46 (metastatic control mediator), NKG2D (NK cell marker) and FoxP3 (T-cell negative regulator) were increased. The author linked this positive regulation of immune system-related pathways with greater activation and infiltration of NK cells into the tumor, which suppressed its growth. In the same study, an increase in inflammatory markers such as IL-1β, IL-6, iNOS, and TNFα was also observed in the tumor of voluntary runners (Bay et al., 2017).

Tumor progression in C57bl/6 mice with LLC-induced cachexia and practicing high intensity interval training through treadmill running was analyzed in a recently published work. It has been observed not only a reduction in tumor volume, but also a higher expression of CD274 (PD-L1), a transmembrane protein responsible for suppressing the immune response through T cell presence in the tumor of trained animals. VEGFα, an important marker of vascularization, was also increased with training in combination with a tendency to increase IL-6 and TNFα expression (Christiano et al., 2018; Table 1).

Hypoxia and lack of blood supply promote an aggressive cancer phenotype, which contributes to the inefficiency of systemic therapy (Shannon et al., 2003). In contrast, vascular normalization, for example, stimulation of vascular endothelial growth factor (VEGF), improves chemotherapy response by improving oxygenation and consequently increased access of drugs to the tumor. Aerobic exercise stimulates a more “normalized” tumor microenvironment by improving intratumoral perfusion/vascularization, as demonstrated in animal models of breast and prostate tumors (Ruiz-Casado et al., 2017).

The mechanisms postulated for the potential effects of exercise on cancer progression include metabolic modulation through evaluation of host glucose-insulin homeostasis and modulation of sex hormones (testosterone), improvements in immune surveillance, and reduction of systemic inflammation and oxidative damage (Jones et al., 2012a). The comprehension of the mechanisms elicited by RT involved in tumor growth control are still at an early stage of development and understanding since the heterogeneous nature of the tumors and the biological variation of the host are challenging variables (Claudia et al., 2017).

## Physical Exercise and Survival

Studies evaluating the effects of exercise training on the survival of patients diagnosed with CC are still scarce. A search of PubMed’s databases was performed by crossing the terms: survival, cancer and exercise, selecting only clinical studies conducted in the last 10 years. With these descriptors a total of 178 papers were found. However, when the search was performed with the replacement of the descriptor “cancer” by “cancer cachexia,” with the same parameters as before, only three studies were found, available in Table 2. In addition, a recent Cochrane review showed that there are no randomized control studies that have addressed exercise interventions in CC (Grande et al., 2014). Notwithstanding, only one of these studies (Solheim et al., 2017) had as inclusion criteria the diagnosis of cachexia according to the international consensus diagnosis and classification of cachexia (Fearon et al., 2011).

**TABLE 2:** Studies published in the last 10 years with cancer patients.

**Author**	**Subjects**	**Cachexia markers**	**Exercise training protocol**			**Main results**
			**Type**	**Protocol**	**Volume/intensity**	
Grote et al. (2018)	Oncologic patients with diagnosed cachexia	↓Body weight ↓Functional capacity	Progressive resistance training	13 training sessions, 3 times weekly for 30 min	3 exercises for major muscle groups with 8–12 repetition maximum	• Improvement of weight loading • Improvement in general fatigue and quality of life in the intervention group
Solheim et al. (2017)	Oncologic patients with diagnosed cachexia	Body mass index < 30 kg/m^2^; and < 20% weight loss in the previous 6 months	Multimodal treatment: anti-inflammatory drug; Eicosapentaenoic Acid (EPA); Nutritional counseling; and Exercise program including home-based aerobic and resistance training	Aerobic exercise as patients’ choice and resistance training	AT for 30 min a Day, 2 times a week; RT consisted in six individualized exercises, three times weekly for about 20 min	• No statistically significant effect on physical activity or muscle mass • Survival was similar between the groups
Rogers et al. (2011)	Oncologic patients with diagnosed cachexia	↓Body weight ↓Functional capacity	Resistance Training + EPA or Cox-2 Inhibitor	20 weeks of RT. 5-10 min warm up, followed by the exercise prescription, and a 5 min cool-down	Not mentioned	• Increase in grip strength • Increase in body weight • Improved levels of fatigue • Decreasing CRP and interleukin-6

*EPA, eicosapentaenoic acid; COX-2, cardiomyocyte cyclooxygenase-2; CRP, C-reactive protein.*

The unique study investigated the effect of multimodal treatment consisting of anti-inflammatory pharmacological treatment, use of EPA-enriched hyperproteic and hyperproteic supplement (ProSure^©^ Abbott), nutritional counseling, and exercise prescription to be performed outside the hospital setting in patients with liver and pancreas cancer during anti-cancer treatment. This was a randomized phase II study, and the results were similar between groups for muscle mass, grip strength, inflammatory, and fatigue parameters. The authors linked these factors to poor treatment adherence (final *n* = 25) (Solheim et al., 2017). Besides, it should be noted that several studies are showing a positive effect of exercise training on the survival of cancer patients, without the presence of cachexia (Holmes et al., 2005; Liu et al., 2006; Holick et al., 2008; Irwin et al., 2008).

Besides, three different experimental model studies using rats or mice, published in the last 10 years, were found. Those experimental models have evaluated the effect of different exercise protocols in the presence of induced cachexia, as available in Table 1. Wistar rats with Walker 256 tumor-induced cachexia submitted to RT (climbing), presented a reduction in survival compared to sedentary tumor animals (Das Neves et al., 2016). The tumor was inoculated into the brown bone marrow and induced a severe reduction in muscle content and physical function that was positively correlated with poor survival. In another study, voluntary running was used as an intervention in C26 tumor-inoculated female BALB/c mice; the presence of running wheels increased the survival of tumor-bearing mice, however, this positive affect was not attributed to environmental enrichment (Pigna et al., 2016). Finally, when a High Intensive Interval Training (HIIT) treadmill training was performed by C57bl/6 Tumor bearing mice, animals presented an increased survival, and reduced tumor volume (Christiano et al., 2018).

The real contribution of exercise and its impact on the life expectancy of patients diagnosed with CC remain an obscure field. However, the numerous local and systemic benefits of exercise are already being clarified (Ruiz-Casado et al., 2017). Exercise is recognized to trigger the formation and secretion of several muscle cytokines, including IL-6, which increases insulin sensitivity and reduces the production of pro-inflammatory cytokines (Grande et al., 2014). Chronically, exercise has a global anti-inflammatory effect, which has been observed in healthy people and early stage cancer patients (Betof et al., 2013). Such an effect would be beneficial in CC as levels of systemic inflammation are associated with weight reduction, exercise capacity and survival (Laird et al., 2013).

## Prospects and Limitations on the Use of Exercise in Cachexia

**FIGURE 3:**
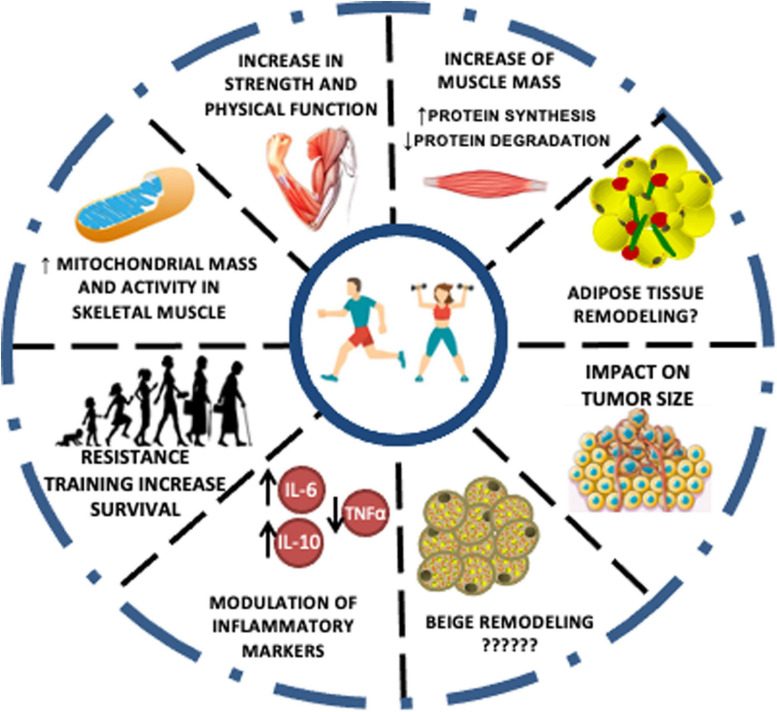
Effects of exercise training in cachexia markers. The effects of different physical training types on cachexia symptoms and markers are evident in the literature; however, in a majority way through tests on experimental models. The changes promoted in skeletal muscle and inflammation control are highlighted as the leading promoters of a better prognosis and increased life expectancy elevated by exercise training.

Exercise training comprises a critical strategy to be used as therapy CC. The exercise effect is related to supporting protein synthesis and muscle growth, resulting in muscle strengthening and improved physical performance. Besides, exercise induces an anti-inflammatory response, potentially abrogating catabolic effects, the primary marker of muscle wasting. In this context, the most suitable modality is based on resistance exercises. Figure 3 illustrates a synthesis of all the events promoted by the different types of exercise against CC up to date mentioned in the literature. Protein synthesis is stimulated to a higher extent than the proteolysis observed after the acute exercise session, which contributes to the protein balance. However, as mentioned above, inflammation control (reduction) is a crucial need for patients with CC. In this scenario, the pivotal role of exercise to stimulate the release of a plethora of cytokines, mainly by the skeletal muscle, so-called myokines, must be considered. The main responses observed as a result of the action of myokines are their ability to improve glucose uptake by muscles, muscle lipolysis and fat oxidation, and thus, mobilize energy reserves. In the case of IL-6, which is the predominant cytokine produced in response to exercise, it is indicated that chronically, after exercise, it is responsible for increasing insulin sensitivity and reducing pro-inflammatory cytokine production. Endurance exercise, in this case, is indicated as a safe and efficient alternative for the control of inflammation observed in patients with CC, considering that the cardiovascular condition is compatible with the prescribed exercise load. Taken together, a proper training prescription should contain the combination of strength and endurance work to meet therapeutic needs. The challenges regarding cancer patients’ adherence to the exercise programs are primarily related to their fatigue tolerance to exercise. Patients with lower baseline fatigue scores and greater adherence to the intervention had the greatest improvements. It is worth noting that patients who previously had lower cancer-related fatigue scores have a greater ability to tolerate exercise and therefore better outcomes (Puetz and Herring, 2012). However, a study of patients undergoing exercise during chemotherapy treatment indicated that as exercise exposure increased the intensity of cancer-related fatigue decreased at all basal levels of fatigue. The authors also highlighted that despite the advanced stage of the tumor and the costly treatment, adherence to the intervention was excellent. Progressive resistance training in patients with head and neck cancer cachexia during radiotherapy proved to be safe and its effects are beneficial in the face of fatigue and quality of life (Grote et al., 2018). Another study indicated that progressive resistance training in cancer patients with cachexia is feasible. The training proved to be well tolerated and safe (Grote et al., 2018; Table 2).

The role of exercise training in CC needs further elucidation in the coming years. Studies of exercise interventions in the field are few in number and most concentrated in animal models. In cachexia patients, there are just a few reports of small studies from conference proceedings as well as ongoing/planned studies. It demands additional randomized trials to investigate exercise training either alone or in combination with pharmacologic treatment. Different variables were indicated as determinants for adherence to the exercise program, such as location of the training center, disease stage, aerobic conditioning, and depression (Schwartz et al., 2001). Given this potential, the major challenge in the applicability of an exercise training program in cancer patients comprises a number of variables such as: (1) prescription aligned according to the proper diagnosis of the cachexia stage in which the patient is; (2) patient motivation for adherence and permanence in the program; and (3) adequate and constant control of training variables so as not to exceed the patient’s physical condition.

## Author Contributions

MB, LL, and ML conceived the review and wrote the manuscript. LL, ML, and SP analyzed the data. All authors contributed to the article and approved the submitted version.

## Conflict of Interest

The authors declare that the research was conducted in the absence of any commercial or financial relationships that could be construed as a potential conflict of interest.
